# High Q-Factor Hybrid Metamaterial Waveguide Multi-Fano Resonance Sensor in the Visible Wavelength Range

**DOI:** 10.3390/nano11061583

**Published:** 2021-06-16

**Authors:** Hongyan Yang, Yupeng Chen, Mengyin Liu, Gongli Xiao, Yunhan Luo, Houquan Liu, Jianqing Li, Libo Yuan

**Affiliations:** 1 School of Electronic Engineering and Automation, Guilin University of Electronic Technology, Guilin 541004, China; yhy.gl@126.com (H.Y.); 19082203004@mails.guet.edu.cn (Y.C.); 19082203009@mails.guet.edu.cn (M.L.); lbyuan@guet.edu.cn (L.Y.); 2 Guangxi Key Laboratory of Automatic Detecting Technology and Instruments, Guilin University of Electronic Technology, Guilin 541004, China; 3 Guangxi Key Laboratory of Precision Navigation Technology and Application, Guilin University of Electronic Technology, Guilin 541004, China; xiaogl.hy@guet.edu.cn; 4 College of Science & Engineering, Jinan University, Guangzhou 510632, China; luoyunhan@jnu.edu.cn; 5 Guangdong-Hong Kong-Macao Joint Laboratory for Intelligent Micro-Nano Optoelectronic Technology, Macau University of Science and Technology, Avenida Wai Long, Taipa, Macau 999078, China; jqli@must.edu.mo

**Keywords:** Fano resonance, hybrid metamaterial waveguide, visible wavelength range

## Abstract

We propose a high quality-factor (Q-factor) multi-Fano resonance hybrid metamaterial waveguide (HMW) sensor. By ingeniously designing a metal/dielectric hybrid waveguide structure, we can effectively tailor multi-Fano resonance peaks’ reflectance spectrum appearing in the visible wavelength range. In order to balance the high Q-factor and the best Fano resonance modulation depth, numerical calculation results demonstrated that the ultra-narrow linewidth resolution, the single-side quality factor, and Figure of Merit (FOM) can reach 1.7 nm, 690, and 236, respectively. Compared with the reported high Q-value (483) in the near-infrared band, an increase of 30% is achieved. Our proposed design may extend the application of Fano resonance in HMW from mid-infrared, terahertz band to visible band and have important research value in the fields of multi-wavelength non-labeled biosensing and slow light devices.

## 1. Introduction

Localized surface plasmon resonance (LSPR) is a resonance phenomenon caused by the collective oscillation of free electrons on the surface of precious metal nanoparticles under the action of photons. Supported by metal nanostructures in the sub-wavelength range, LSPR has a large local electromagnetic field enhancement effect [1,2], to be applied to surface-enhanced Raman scattering [3,4], electromagnetic induction transparency (EIT) [5,6,7], and in other fields. However, its quality factor (Q-factor) is relatively low (Q < 10) [8,9] to achieve ultra-narrow-band resonance due to the excessively high ohmic loss of the metal, thus resulting in the impracticality of potential applications based on surface plasmon resonance. In recent years, a large number of studies have extensively conducted and deeply explored the optical nanodevices that excite ultra-high Q resonance lines to overcome this defect, mainly focusing on: the resonators of high refractive index dielectric materials related to bound or quasi-bound states in the continuum excite the Fano resonance of high Q-factor through strong coupling between modes [10,11,12,13,14,15], and the plasma lattice resonance and Fano resonance based on periodic structure [8,16,17,18,19,20,21]. Researchers found that the bound state (BIC) in the continuum can be confined completely, without any radiation, and reach a high Q-value (10^4^) [10,11,12,13], and the all-dielectric material not only eliminates the ohmic loss, but also the radiation loss of the magnetic response is low, and devices that respond through multipole high-Q resonance in the near-infrared band have been rapidly developed [17,22]. Recent experiments proved that based on plasma-related surface lattice resonance, a Q factor greater than 2000 can be achieved [16,23]. In addition, the introduction of Fano resonance is effective to achieve ultra-narrow-band resonance [20,21,24,25], and it shows stronger tolerance to uneven environments, which have great potential in far-field emission applications. Supported by metal nanostructures, the Fano resonance is formed by super-radiation states (bright mode) and sub-radiation states (dark mode) superimposed in the spectral range to produce interference [26,27]. Compared with the symmetrical Lorentz curve, the asymmetric Fano resonance is formed by the coupling of discrete bound states and wide continuous states. During the coupling process, the Fano resonance spectrum curve not only has obvious asymmetry and phase mutation, but it will also be sensitive to changes in structural parameters and the refractive index of the surrounding environment. Therefore, in recent years, high Q nanostructured devices based on Fano resonance have become a research hotspot in the fields of biochemical sensing, optical communication, on-chip photonics, and nonlinear optics [28,29,30,31].

In 2013, Shen et al. successfully demonstrated a near-infrared (NIR) mushroom-style Fano resonance biosensor. An asymmetric Fano formant is achieved through the coupling of LSPR and Wood anomaly, with a FOM of 108 [32]. Following this, there was great enthusiasm in the topic and in the design of the metamaterial structure of periodic metal nanoarrays [33,34,35,36]. In 2017, Lee’s group generated Fano resonance by using the interaction between cavity resonance in nanowires (continuous state) and Wood anomaly/Bloch wave surface plasmon (discrete resonance state) by designing periodic aluminum/medium and aluminum/substrate interface structures. The high-sensitivity aluminum-based biosensor in the visible light wavelength range is experimentally studied. The intensity sensitivity of the sensor can reach 29,345.05%/RIU [33]. In 2018, Chen et al. focused on the dielectric flat waveguide composed of a two-dimensional gold nanoparticle array, and under the condition that the electric field in the waveguide mode was parallel to the polarization direction of LSPR, Fano resonance was achieved in the near-infrared spectra (NIR), and the sensitivity can reach 250 and 200 nm/RIU [37]. In 2020, He’s group investigated the lattice array of Au/TiO_2_ mixed meta-surface on a Si-based surface by coupled mode theory in the visible wavelength band, and realized the Fano resonance between surface plasmon polariton and LSPR. Modulation of the lines and depths of multiple Fano resonances are found to be achieved by adjusting the size and location of the short bar defects, with the results of the sensitivity reaching 330 and 535 nm/RIU [38].

To sum up, three types of structures to achieve Fano resonance have been largely focused on: metal surface plasmon structure [18,39,40], hybrid metamaterial waveguide (HMW) structure [37,41,42], and all-dielectric structure [6,22,43]. A large number of studies show that the Fano resonance, excited by constructing symmetrical broken metal structures in the terahertz band, is sensitive to the external environment, though its Q-factor is usually low. Due to the extremely low electromagnetic loss in the all-dielectric structure and the inherent characteristics of BIC, an extremely high Q-factor can be obtained in the near-infrared band. The HMW plasmon resonant structure can not only achieve high quality-factor resonance, but also has a high degree of modulation freedom. A lot of theoretical studies have been carried out, and experimental verification on the manipulation mechanism of Fano resonance in the near-infrared to terahertz band has been carried out. In previous reports, due to the limitations of the ohmic and strong scattering loss of metals in the visible light band [33,38], there are few reports on devices that achieve a high Q-factor in the visible light wavelength range. Obviously, it is still a challenge to obtain high Q-factor multi-Fano resonance peaks in the visible light band and achieve extremely high detection accuracy for sensing applications.

In this study, we propose a type of high Q-factor HMW multi-Fano resonance sensor in the visible light spectrum. Asymmetric linear Fano resonance is realized by designing a sub-wavelength nanoarray unit with a metal cap/dielectric structure, and introducing a high refractive index waveguide layer to excite the LSPR and waveguide mode structure. The strong coupling between the LSPR of the metal array and the different guided modes, which can produce extremely narrow bands of Fano resonance response with high Q-factor, were theoretically proven. The Fano resonant line width, Q-value, and the FOM can reach 1.7 nm, 690, and 236, respectively. This may have great potential in the field of label-free biochemical sensing and narrow-band detection in the visible light band.

## 2. Structural and Theoretical Analysis

### 2.1. Modeling Design

**Figure 1:**
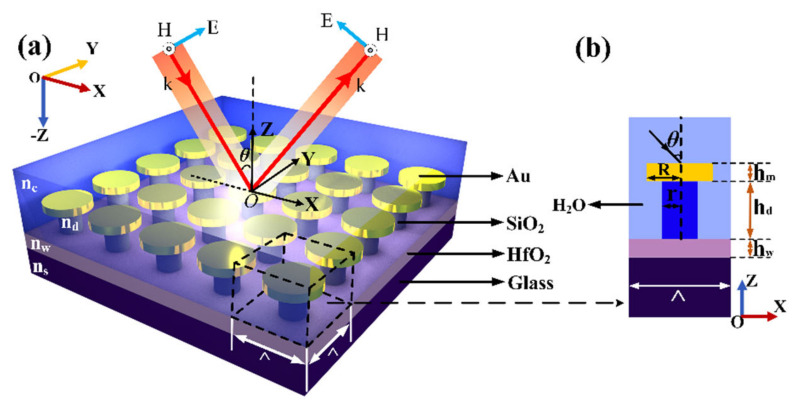
(**a**) Schematic diagram of the “mushroom” array hybrid waveguide structure in an asymmetric medium environment. (**b**) Side view of a single nanoparticle unit (xoz plane).

Figure 1 shows our proposed subwavelength hybrid waveguide nanostructure. We use SiO_2_ pillar to raise the Au disc, which can not only reduce the base effect [44], but also make the metal disc more exposed to the surrounding environment, thereby effectively improving its refractive index sensitivity and reducing its full width at half maxima (FWHM) [45]. In the simulation, the radius and thickness of the Au disc are *R* = 100 nm and *h_m_* = 100 nm, and the radius and thickness of the SiO_2_ pillar are *r* = 80 nm and *h_d_* = 300 nm. The period of the “mushroom” unit along the *x*- and *y*-axis is Λ = 400 nm. The nanopillar array is located on the HfO_2_ waveguide layer with a height of *h_w_* = 100 nm. We use the dielectric function of gold measured experimentally in the literature [46], the refractive index of the SiO_2_ pillar is $n_{d}$ = 1.45, the refractive index of the HfO_2_ waveguide layer is $n_{w}$ = 2, and the refractive index of the lowermost glass substrate is $n_{s}$ = 1.52. Since $n_{w}$ > $n_{s}$ > $n_{d}$, when the dispersion condition of the guided-mode resonant grating is satisfied, a waveguide mode with stable transmission can be formed in the waveguide layer (HfO_2_). Unless otherwise specified, the array structure is immersed in a water environment with a refractive index $n_{c}$ = 1.33. In this structure, a TM polarized wave is obliquely incident from above the structure in the xoz plane, with an incident angle of θ.

### 2.2. The Physical Mechanism

**Figure 2:**
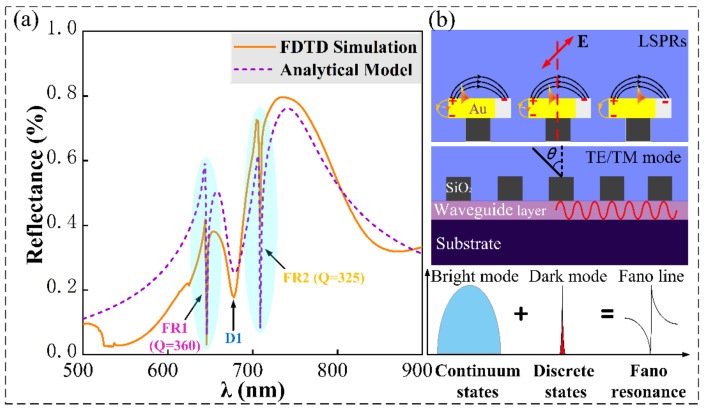
(**a**) FDTD numerical simulation of the reflection spectrum and the reflection spectrum fitted by the harmonic oscillator oscillation model. (**b**) LSPR and waveguide modes are coupled to form a schematic diagram of Fano resonance.

The structure is simulated by using FDTD solutions of Lumerical company’s business software. In the simulation, to ensure the accuracy of the simulation results, the grid of the effective simulation area is set to 2 nm. The perfect matching layer (PML) boundary conditions are set in the z direction, and the periodic boundary conditions are set in the x and y directions. In our simulations, the TM polarization wave along the *z*-axis inclined the incidence. Next, we use the finite difference time domain method to numerically simulate the optical response of the structure in the visible band. Numerical simulation results are shown by the solid line in Figure 2a. It can be seen that two Fano resonance peaks with a high Q-factor appear at 645.8 (FR1) and 709 nm (FR2). Here, we introduce the single side Q-factor and FOM calculation formulas of Fano resonance [47]. Q-factor can be expressed as: Q_ss_ = 0.5 × λ_min_/(λ_min_ − λ_hp_), and FOM is expressed as: FOM = Q × ∆I. Among them, the Q-factor and resonance intensity are two important parameters for evaluating the quality of the formant. $\lambda_{\min}$ represents the wavelength at minimum power, $\lambda_{\mathrm{hp}}$ represents the wavelength at half power, and ΔI is the resonance intensity. The Q-factor of FR1 is 360, and the Q-factor of FR2 is 325, as shown in Table 1. In addition, there is a Fano linear resonance valley with a lower Q-factor at 677.6 nm (D1).

As shown in Figure 2b, when the TM polarized wave incident is oblique, the incident light (**E_0_**) can be orthogonally decomposed into **E_x_** and **E_z_**. Among them, the dipole distribution of the gold disk caused by **E_x_** is called the in-plane dipole mode, and most of the energy scattered by the in-plane dipole mode can be radiated into free space, so it can be regarded as a bright mode (super-radiation mode); similarly, the dipole distribution caused by **E_z_** is called the out-of-plane dipole mode. The out-of-plane dipole mode can effectively suppress the radiation attenuation, so it can be regarded as the dark mode (sub-radiation mode) [48]. In addition, since the periodic array can provide the necessary energy to couple the diffracted waves into the waveguide layer, narrow-band TE- and TM-guided wave modes can be excited. These guided wave modes can be regarded as dark modes (sub-radiation states). Therefore, the physical origin of FR1, D1, and FR2 can be explained by the coupling between harmonic oscillators with different damping coefficients. The in-plane dipole mode with large radiation loss can be regarded as a harmonic oscillator with a large damping coefficient. The low radiation loss in the in-plane dipole mode and the waveguide mode in the waveguide layer can be regarded as harmonic oscillators with a small damping coefficient. FR1, D1, and FR2 are respectively formed by the coupling of the above-mentioned resonator with a larger damping coefficient and a resonator with a smaller damping coefficient. So, in the simulation results, FR1 and FR2 can be regarded as Fano resonance modes, in which the sub-radiation mode (waveguide mode) is coupled to the super-radiation mode (plasma dipole mode), and destructive interference causes the spectrum to drop sharply and then rise. D1 can be considered as a strong coupling between the plasmon dipole mode with a wide line width and the dipole moment of the out-of-plane nanoparticle, thereby suppressing the attenuation of radiation and presenting a sub-radiation phenomenon similar to the Fano line.

## 3. Results and Discussion

### 3.1. Far Field and Near Field

In order to more clearly understand the causes of the formation of various resonance peaks and valleys, we first studied the far-field reflection spectrum and the near-field electric field distribution of the x-polarized wave at normal incidence (*θ* = 0°) and oblique incidence (*θ* ≠ 0°) without the presence of the waveguide layer. Finally, the far-field reflection spectrum and the near-field distribution of the x-polarized wave are comparatively studied when the x-polarized wave is obliquely incident, and the waveguide layer is present.

**Figure 3:**
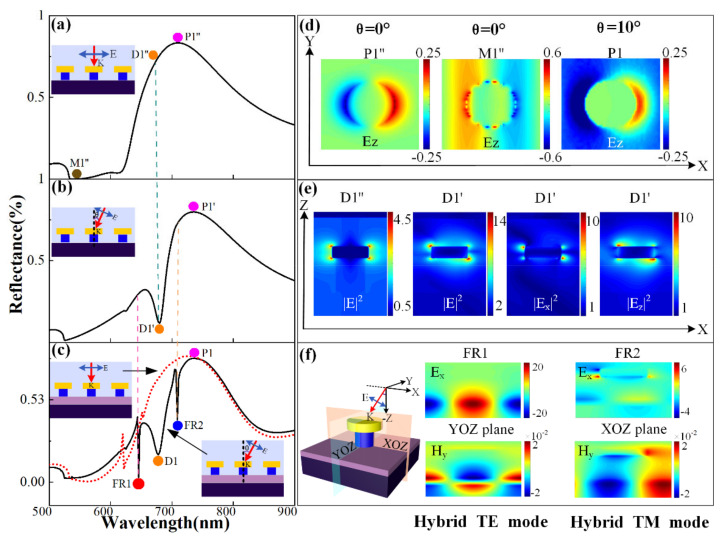
(**a**) The reflectance spectrum when the light source is normal incident and when there is no waveguide layer. (**b**) The reflectance spectrum when the light source is obliquely incident and there is no waveguide layer. (**c**) The reflection spectrum of the light source at normal incidence and oblique incidence, when there is a waveguide layer. (**d**) In the xoy plane, the z component of the electric field (**E_z_**) at normal incidence (P1′’, M1′’) and oblique incidence (P1). (**e**) In the xoz plane, the comparison of the ${\left|E\right|}^{2}$ at the D1′’ position at normal incidence and the D1′ position at oblique incidence, and the ${\left|E_{x}\right|}^{2}$ and ${\left|E_{z}\right|}^{2}$ components of the D1′ position at oblique incidence. (**f**) The distribution of the electromagnetic field components $E_{x}$ and $H_{y}$ of the waveguide TE mode on the yoz plane and the distribution of the electromagnetic field components $E_{x}$ and $H_{y}$ of the waveguide TM mode on the xoz plane.

As shown in Figure 3a, when there is no waveguide layer and the light source polarized along the x-direction is perpendicularly incident, the LSPR supported by the metal nanoarray form a resonance peak (P1″) with a FWHM far greater than 100 nm at 709 nm. The electric field distribution $\left(E_{z}\right)$ is shown in Figure 3d. When the light source is obliquely incident, it presents an asymmetric Fano-like tilt angle (D1′) at 678 nm, as shown in Figure 3b. Next, after maintaining the oblique incidence and adding the waveguide layer, we found that in Figure 3c, two Fano resonance peaks (FR1 and FR2) with extremely narrow linewidths appeared at 645.8 and 708.8 nm. To clarify the reasons for the formation of D1, FR1, and FR2, we continued to analyze their electromagnetic field (E and H) near-field distribution. By observing the electric field component at D1′, we can determine that ${\left|E_{x}\right|}^{2}$ is the in-plane lattice resonance mode of the metal cap array driven by the in-plane dipole oscillation, and ${\left|E_{z}\right|}^{2}$ is the out-of-plane dipole mode, which is oscillated by the out-of-plane dipole to drive [48]. Comparing the near-field electric field distribution, it is found that the strong coupling of the out-of-plane dipole oscillation and the in-plane dipole oscillation of the nanoparticle array can effectively capture the incident light and cause a large field enhancement in and around the array plane. Due to the interference between the narrow (sub-radiation) out-of-plane plasmon resonance and the wide (super-radiation) in-plane plasmon resonance, the strongly coupled metal array shows a Fano-type asymmetric spectral profile. By observing the electromagnetic field distribution near the waveguide layer at FR1 and FR2, as shown in Figure 3f, we can find that there is a stable transmission mode in the waveguide at the two Fano resonance peaks. Therefore, we study the significance of the waveguide layer to explore the observed Fano line’s mechanism.

Next, we need to further discuss the inverted mode of the slab waveguide. The waveguide layer is located between the substrate and the background environment and can be considered as a slab waveguide supporting TE mode and TM mode. According to the slab waveguide theory, the guided mode should satisfy the energy dispersion equation [49]:

(1) $$\tan\left(kh_{w}\right)=\frac{k\left(\gamma_{c}+\gamma_{s}\right)}{k^{2}-\gamma_{c}\gamma_{s}},\;\;(\mathrm{TE}\;\mathrm{mode}),$$

(2) $$\tan\left(kh_{w}\right)=\frac{k\left(\frac{\gamma_{c}n_{w}{}^{2}}{n_{c}{}^{2}\gamma_{c}}+\frac{\gamma_{s}n_{w}{}^{2}}{n_{c}{}^{2}\gamma_{c}}\right)}{k^{2}-\frac{n_{w}{}^{4}}{n_{c}{}^{2}n_{s}{}^{2}}\gamma_{c}\gamma_{s}},\;\;(\mathrm{TM}\;\mathrm{mode}),$$

In the formula, $k=k_{0}\sqrt{n_{w}^{2}-n_{eff}^{2}}$, $\gamma_{c}=k_{0}\sqrt{n_{eff}^{2}-n_{c}^{2}}$, $\gamma_{s}=k_{0}\sqrt{n_{eff}^{2}-n_{s}^{2}}$, $k_{0}=\frac{2\pi }{\lambda }$, where $k_{0}$ is the propagation constants in the xoy plane, and $n_{eff}^{}$ is defined as the effective refractive index of the waveguide mode. According to Equations (1) and (2), when $n_{c}$ = 1.33, $h_{w}$ = 100 nm, and $n_{w}$= 2, we can obtain the dispersion relationship between TE and TM modes. When the periodic metal cap/dielectric array structure is deposited on the dielectric plate waveguide, it can provide the necessary power to couple diffracted waves into the waveguide layer to excite the guided mode. In our design, the energy required to excite the guide mode is provided by a two-dimensional metal nanoparticle array with a period of 400 nm. In order to achieve effective resonant coupling, the phase matching conditions need to be met:

(3) $$\beta_{WG}=n_{eff}k_{0}=k_{x-y}\pm m\frac{2\pi }{P_{x}}x\pm n\frac{2\pi }{P_{y}}y$$

Among them, $k_{x-y}$ represents the component of the incident wave vector in the x-y plane, m and n are integers, representing the order of grating vectors on the *x*-axis and *y*-axis respectively, and $P_{x}$ and $P_{y}$ are the array periods in the x and y directions, respectively.

When the light is obliquely incident on the metal/dielectric periodic array structure, the metal disc is used as an electric dipole antenna, directly coupled with the excitation of the free space to form a plasmon mode (bright mode), and along with the x directional oscillation, we can see the dipole mode in the electric field distribution of metal nanoparticles in Figure 3d. In addition, due to the oblique incidence of polarized waves along the x direction and the periodic array of metal nanoparticles providing the necessary momentum to excite the TE and TM modes, a large amount of electromagnetic wave energy concentrates around the waveguide layer. From Figure 3f, we can intuitively determine that the TE mode exists at the FR1 position, which propagates in the y direction, and the wavelength of FR2 supports the excitation of the TM mode, which extends in the x direction. Since the waveguide mode cannot be directly excited by the incident plane wave, it can be regarded as a plasmon dark mode. Therefore, we believe that FR1 and FR2 are destructive interference between the dipole mode (super-radiation mode) and the waveguide mode (sub-radiation mode) excited by the metal nanoparticles, which leads to the excitation of the Fano resonant, as shown in Figure 3c.

### 3.2. Structural Parameter Analysis

**Figure 4:**
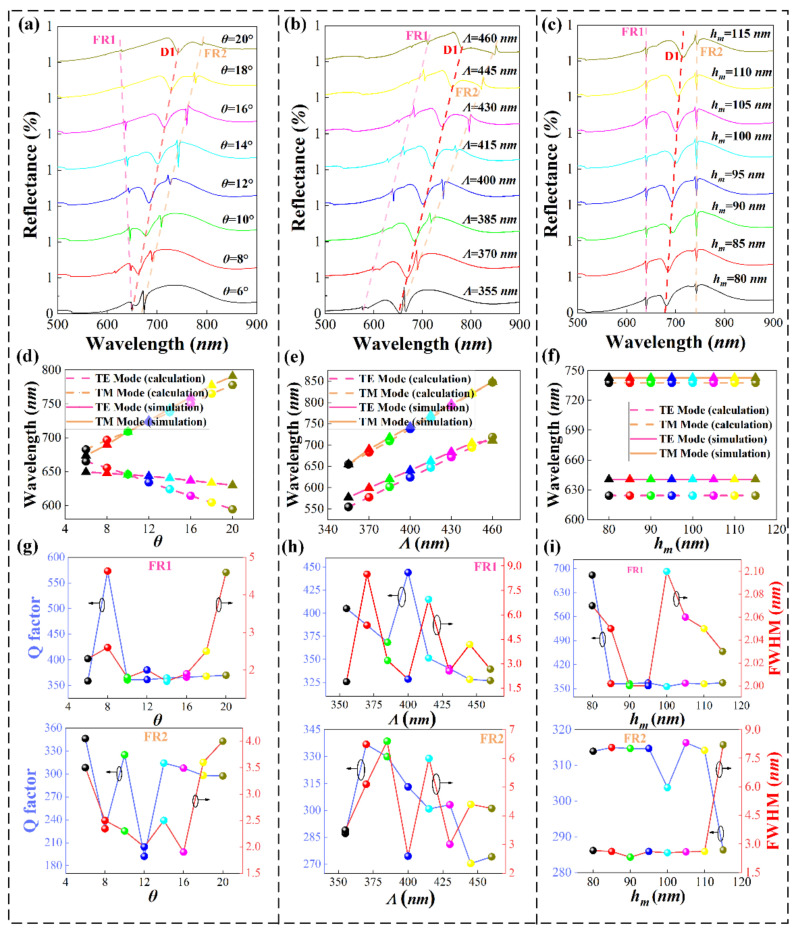
(**a**) The reflection spectrum of the nanoarray at different incident angles, (**b**) the reflection spectrum of the nanoarray at different periods, and (**c**) the reflection spectrum of the nanoarray at different Au disc thickness. The position of the TE and TM waveguide modes of the nanoarray at different (**d**) incident angles, (**e**) periods, and (**f**) Au disc thickness. The Q-factor and FWHM of FR1 and FR2 at different (**g**) incident angles, (**h**) periods, and (**i**) Au disc thickness.

Next, we found that the two-dimensional nanoarray-waveguide coupling system in an asymmetric medium environment can be dynamically tuned by changing the incident angle and statically tuned by changing the geometric parameters, as shown in Figure 4.

Firstly, as shown in Figure 4a, we explored the increase in the incident angle θ from 6° to 20°, while maintaining the reflection spectrum changes of Λ = 400 nm and $h_{m}$ = 100 nm. It is not difficult to find that FR1 has a blue shift and FR2 has a red shift. To explain this phenomenon, we calculated the TE/TM mode wavelength positions supported by the slab waveguide at different angles through the above Equations (1)–(3), as shown in Figure 4d. By comparing Figure 4a,d, we can see that as the angle increases, the TE mode supported by the waveguide layer shifts to blue, and the TM mode shifts to red. The LSPR (P1) also has a red shift phenomenon. The coupling degree of the TE waveguide mode and LSPR gradually decreases until FR1 disappears. The TM waveguide mode and LSPR wavelength are red-shifted at the same time, and the FR2 resonance peak formed by coupling is red-shifted. It can be seen from Figure 4g that the Q and FWHM of FR1 at 14° are 364 and 1.7 nm respectively, and the corresponding FOM value is 98, while the quality factor and FWHM of FR2 are 314 and 2.5 nm respectively, and the FOM value is 236. Similarly, as the period increases from 355 to 460 nm, while maintaining θ=14° and $h_{m}$= 100 nm, Figure 4b shows the effect of different periods on the reflection spectrum of the two-dimensional nanoarray-waveguide coupling system. In conjunction with Figure 4e, the TE/TM modes are all red-shifted, and LSPR also moves to the long-wave direction, resulting in the FR1 and TM modes shown in Figure 4b. As the period increases, an FR2 red shift occurs. In addition, combined with the numerical analysis of Figure 4h, when the period is 400 nm, the Q-factor and FWHM of FR1 can reach 444 and 2.05 nm, and the corresponding FOM is 155, while the Q-factor of FR2 and FWHM can reach 313 and 2.595 nm, and the corresponding FOM is 171. Finally, as shown in Figure 4c, we show the effect of changing the height, $h_{m}$, of the metal nanoarray on the Fano resonance peak. As the thickness of the metal cap changes, the resonance valley intensity of FR1 and FR2 changes, and the wavelength position does not change. This is because the change of the thickness of the gold cap will not affect the waveguide mode, but the change of the size of the gold cap will cause the shift of the wavelength position of the local surface plasmon resonance peak, which will cause the FR1 and FR2 resonance intensities to change. Besides, Figure 4i shows that when the height of the nano-metal particle array, $h_{m}$, is 105 nm, the maximum Q-value of FR1 is 366, and the corresponding FWHM is 2.06 nm, while the Q value of FR2 is 316 and FWHM corresponds to 2.58 nanometers. The results of the above parameter optimization studies show that our results are several times higher than those in similar published studies in visible wavelength band [33,38].

Besides, it can be seen from Figure 4g–i that when the incident angle, period, and the thickness of the gold disk change regularly, the Q-factor of the numerical simulation fluctuates greatly. Therefore, we significantly reduced the angle step size for comparative simulation, and the calculation results are shown in Table 2. We can see that Q-factor remains stable under the change of small step length. Finally, we used the Fano parameter q (q=cot(δ)) to explain the phenomenon of fluctuation of the Q-factor, where q represents the relative excitation intensity associated with the discrete and continuous states, and δ is the phase difference between the two modes. The phase difference between the LSPR and the waveguide mode can be changed by changing the incident angle, the period, and the thickness of the gold disc. In addition, the variation of structural parameters will affect the degree of coupling between the two modes, or the coupling may not even occur. Finally, it changes the resonance lines, affecting the Q-factor of the formants [50,51].

### 3.3. Influence of Dielectric Environment

**Figure 5:**
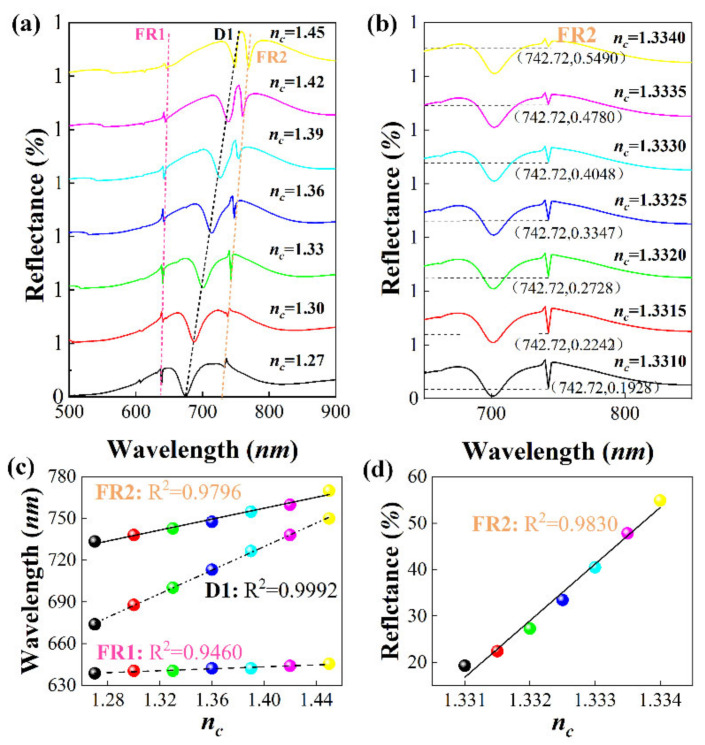
(**a**) Reflectance spectrum of nanoarray with refractive index (1.30~1.45). (**b**) Reflectance spectrum of nanoarray with refractive index (1.3310~1.3340). (**c**) Nanoarray refractive index (1.30~1.45) combined sensitivity simulation. (**d**) Fitting figure of the magnitude of the refractive index (1.3310~1.3340) of the nanoarray.

High Q-factor and extremely narrow linewidth can be expected for sensing. Finally, we discuss the wavelength sensitivity and amplitude sensitivity of the sensor. The wavelength sensitivity can be expressed as: S=dλ/dn; similarly, the amplitude sensitivity can be expressed as: S=dI/dn, where dn represents the change of refractive index, dλ represents the change of resonance wavelength, and dI represents the change of resonance valley amplitude. Figure 5a shows the variation of FR1, D1, and FR2 resonance peaks with the refractive index when the environmental refractive index increases from 1.27 to 1.45. As the refractive index increases, FR1, D1, and FR2 show a consistent red shift. We think this is caused by the red shifts of TE/TM mode and the resonance position of LSPR. Figure 5b shows the change of resonance dip amplitude corresponding to FR2 with the refractive index when the environmental refractive index increases from 1.3310 to 1.3340. We attribute the change in FR2 resonance intensity to the different phase between the TM mode (dark mode) supported by the waveguide and LSPR (bright mode).

Next, as shown in Figure 5c,d, by fitting the wavelength sensitivity curve of FR1, D1, and FR2, and the intensity sensitivity curve of FR2, we obtain the following formula:

(4) $$\left\{\begin{array}{l} \lambda_{FR1}\left(nm\right)=35.06n_{c}+594.26,1.27\leq n_{c}\leq 1.45\\ \lambda_{D1}\left(nm\right)=422.38n_{c}+138.34,1.27\leq n_{c}\leq 1.45\\ \lambda_{FR2}\left(nm\right)=196.12n_{c}+482.67,1.27\leq n_{c}\leq 1.45\\ \lambda_{FR2}\left(\%\right)=12200.57n_{c}-16222.17,1.3310\leq n_{c}\leq 1.3340 \end{array}\right.$$

Finally, as shown in Table 3, through comparison with previous work, the designed tunable ultra-narrow-band Fano resonant plasma sensor not only has good sensing performance in the visible light wavelength range, but also has extremely high detection sensitivity.

## 4. Conclusions

In summary, in this paper, we proposed a tunable ultra-narrow-band Fano resonance plasmonic sensor, and conducted numerical and theoretical studies on it. A sharp and asymmetric Fano distribution was observed in the reflection spectrum, which originated from the interaction between the LSPR and the different guided modes of the waveguide. The incident angle and structural parameters of the TM polarized wave can adjust the line type and position of the Fano resonance, especially for changes in the incident angle θ, structural period, and environmental refractive index parameters. The proposed sensor can achieve an extremely narrow Fano resonance linewidth of 1.7 nm in the visible light wavelength range, with a Q-factor of up to 690 and FOM of up to 236. The research results provide a theoretical basis for the design of narrow-band, high Q-factor optical devices in the visible light band, and show great development potential in the next generation of plasmon high-resolution, label-free biochemical sensing, narrow-band detection fields.

## Figures and Tables

**Table 1:** Q-factor and FOM values around Fano resonances.

	$\lambda_{min}(nm)$	$\lambda_{hp}(nm)$	Q	ΔI	FOM
FR1	645.790	644.895	360	0.387	139
FR2	708.771	707.681	325	0.324	105

**Table 2:** Q-factor changes as the incident angle changes.

*θ* (Step is 0.05°)		13.90	13.95	14.00	14.05	14.10
Q	FR1	364	368	364	363	376
	FR2	320	317	314	314	315

**Table 3:** Comparison of the device performance with previous work.

Mechanism	Q	Waveband	FWHM (nm)	Structure	S (nm/RIU)	Ref.
EIT	483	Near infrared	---	All-dielectric ring bar	289	[6]
EIT	139	Near infrared	---	Perpendicular bar	294	[30]
SLR	147	Near infrared	4.8	MIM lattice array	368	[9]
SLR	2340	Near infrared	0.66	Au array	---	[23]
Fano	9700	Near infrared	---	All-dielectric pillars	344	[22]
Fano	196	Near infrared	---	Au Ring/Rod Metasurface	---	[47]
Fano	23.4	Visible light	---	Dielectric/metal array	535	[38]
Fano	---	Near infrared	---	Dielectric waveguide	250	[29]
Fano	12.8	THZ	---	Plasmonic metasurface	497.8	[16]
Fano	690	Visible light	1.7	Lattice array/Waveguide	196	In this work

## Data Availability

The data is available on reasonable request from the corresponding author.
